# LncRNA SNHG12 promotes cell proliferation and inhibits apoptosis of granulosa cells in polycystic ovarian syndrome by sponging miR-129 and miR-125b

**DOI:** 10.1186/s13048-024-01392-6

**Published:** 2024-04-02

**Authors:** Feilan Xuan, Ruiying Jin, Weimei Zhou, Yongju Ye, Yuefang Ren, Jiali Lu, Aixue Chen

**Affiliations:** 1 Department of Obstetrics and Gynecology, Hangzhou Traditional Chinese Medicine Hospital Affiliated to Zhejiang Chinese Medical University, Hangzhou, Zhejiang 310007 China; 2 Department of Gynecology, Jiaojiang Maternal and Child Health Hospital, Taizhou, Zhejiang 318000 China; 3 Department of Ultrasound, Jiaojiang Maternal and Child Health Hospital, Taizhou, Zhejiang 318000 China; 4 Department of Gynecology, Lishui Hospital of Traditional Chinese Medicine, Lishui, Zhejiang 323000 China; 5 Department of Gynecology, Huzhou Maternity & Child Health Care Hospital, Huzhou, Zhejiang 313000 China; 6 Department of Gynecology, Changxing People’s Hospital of Chongming District, No.1008 Fengfu Road, Changxing Town, Chongming District, Shanghai, 201913 China

**Keywords:** LncRNA SNHG12, Polycystic ovarian syndrome, Granulosa cells, MiR-129, MiR-125b

## Abstract

**Background:**

Polycystic ovarian syndrome (PCOS) is the most common endocrine disease in women of childbearing age which is often associated with abnormal proliferation or apoptosis of granulosa cells (GCs). Studies proved that long non-coding RNA SNHG12 (lncRNA SNHG12) is significantly increased in ovarian cancer and cervical cancer patients and cells. The inhibition of lncRNA SNHG12 restrains the proliferation, migration, and invasion in tumor cells.

**Objective:**

This study explores the role of lncRNA SNHG12 in the apoptosis of GCs in PCOS and the underlying regulated mechanism.

**Methods:**

In this study, the injection of dehydroepiandrosterone (DHEA) successfully induced the PCOS model in SD rats. The human granulosa-like tumor cell line KGN was incubated with insulin to assess the effects of lncRNA SNHG12 on GC proliferation and apoptosis.

**Results:**

Overexpression of lncRNA SNHG12 influenced the body weight, ovary weight, gonadal hormone, and pathological changes, restrained the expressions of microRNA (miR)-129 and miR-125b, while downregulation of lncRNA SNHG12 exerted the opposite effects in PCOS rats. After silencing lncRNA SNHG12 in cells, the cell viability and proliferation were lessened whereas apoptosis of cells was increased. A loss-of-functions test was implemented by co-transfecting miR-129 and miR-125b inhibitors into lncRNA SNHG12-knocking down cells to analyze the effects on cell viability and apoptosis. Next, the existence of binding sites of SNHG12 and miR-129/miR-125b was proved based on the pull-down assay.

**Conclusion:**

lncRNA SNHG12 might be a potential regulatory factor for the development of PCOS by sponging miR-129 and miR-125b in GCs.

**Supplementary Information:**

The online version contains supplementary material available at 10.1186/s13048-024-01392-6.

## Background

Polycystic ovary syndrome (PCOS) is a common cause of female infertility and is characterized by a disorder or loss of ovulation function [1, 2], causing adverse physical and mental effects in female patients. Hirsutism, irregular menstrual cycles, acne, obesity, insulin resistance (IR), and ovarian overreaction are other symptoms of PCOS [3]. The prevalence rate of the disease is approximately 8–13% worldwide [4]. Studies have shown that abnormal follicle development and excessive androgen secretion in PCOS patients are related to abnormal granulosa cells (GCs) [5].

Follicular development is a complex process involving multiple cells and stages. GCs play an important role in oocyte development by providing essential nutrients and growth factors to oocytes [6]. The balance of proliferation and apoptosis in GCs has an extremely important impact on the hormonal microenvironment in the ovary and the formation, growth, and development of the egg and the subsequent embryo [7]. However, the GCs layer around the follicles of PCOS patients showed signs of atresia, degeneration, and hypertrophy, suggesting abnormal proliferation or apoptosis of GCs, which could lead to follicular development and ovulation disorders in PCOS. Therefore, it is important to explore the molecular mechanism of abnormal granulosa cell function to understand the pathogenesis of PCOS.

Long non-coding RNA (lncRNA) is a type of RNA with a length of over 200 nucleotides that does not participate in protein-coding functions [8]. Changes in its activity are closely associated with cell growth, proliferation, differentiation, and apoptosis. An increasing number of studies have shown that lncRNAs are related to PCOS, playing a role in sex hormone secretion, IR, follicle development, GCs proliferation, and apoptosis, and participate in almost every link between the occurrence and development of PCOS [9, 10]. Micro-RNAs (miRNAs) are another type of non-coding RNA, with 19–25 nucleotides in length. miRNAs are involved in the regulation of gene expression at the post-translational level [11]. LncRNAs act as miRNA sponges, which means that they can bind to miRNAs and affect the expression of downstream mRNA through the lncRNA/miRNA axis, thus controlling the occurrence of various diseases [12].

Recent studies have highlighted the significant upregulation of lncRNA SNHG12 in ovarian and cervical cancers [13, 14], where its suppression has been shown to markedly reduce tumor cell proliferation, migration, and invasion. These findings underscore the pivotal role of lncRNA SNHG12 in the pathogenesis of female reproductive system diseases, suggesting its potential as a therapeutic target. Despite these advances, the specific function of lncRNA SNHG12 in human granulosa cells, particularly in the context of PCOS, remains largely unexplored. Furthermore, miR-129 and miR-125b have been identified as potential targets of lncRNA SNHG12 through bioinformatics analysis using StarBase (https://starbase.sysu.edu.cn/agoClipRNA.php?source=lncRNA&flag=target&clade=mammal&genome=mouse&assembly=mm10&miRNA=all&clipNum=1&deNum=0&target=SNHG12), indicating a possible regulatory mechanism. However, the precise nature of the interaction between lncRNA SNHG12 and these miRNAs, and its implications for granulosa cell function and PCOS pathology, have yet to be elucidated. This study aims to bridge this gap by investigating the role of lncRNA SNHG12 in granulosa cells and its potential regulatory relationships with miR-129 and miR-125b, thereby contributing to a deeper understanding of PCOS pathogenesis and identifying novel therapeutic avenues.

This study aimed to explore the influence of lncRNA SNHG12 on apoptosis in granulosa cells within the context of PCOS and to unravel the underlying regulatory mechanisms. The investigation involves the induction of the PCOS model in Sprague-Dawley (SD) rats by administering dehydroepiandrosterone (DHEA). Additionally, the human granulosa-like tumor cell line KGN is subjected to insulin incubation to assess the impact of lncRNA SNHG12 on GC proliferation and apoptosis. Through this comprehensive approach, the study aims to provide insights into the molecular mechanisms governing apoptosis in granulosa cells in the presence of lncRNA SNHG12, contributing to a better understanding of the pathogenesis of PCOS.

## Materials and methods

### In vivo animal experiments

#### Animal adoption

Thirty 6-week-old female SD rats, which were specific pathogen-free (SPF) grade and weighed 170–180 g, were purchased from Shanghai Jihui Laboratory Animal Care Co., Ltd. The animal production license number was SCXK (Hu) 2017-0005. The rearing temperature was 22–25 °C and humidity was 50–80%. The rats were subjected to artificial 12-hour (h)/12 h circadian light. We followed the regulations of the Institutional Animal Care and Use Committee for Animal Experiments. The animal research was approved by the Ethics Committee of the Animal Center of the Zhejiang Eyong Pharmaceutical Research and Development Center. Animal use license number SYXK (Zhe) 2021-0033. Every effort was made to alleviate animal suffering. Adaptive feeding was conducted for a week prior to the experiment.

#### Establishment of PCOS model and group administration

Five rats were retained as the control group and given a normal diet, and the other 25 rats were injected subcutaneously with 6 mg/100 g dehydroepiandrosterone (DHEA) for 21 consecutive days to induce PCOS according to the previous study [15]. After five randomly selected rats were euthanized for model verification, the remaining 20 PCOS rats were randomly divided into four groups: DHEA group, NC group, overexpression lncRNA SNHG12 (oe-lncRNA SNHG12) group, and silencing lncRNA SNHG12 group (si-lncRNA SNHG12) (*n* = 5). The oe-LncRNA SNHG12-Lentivirus was injected intrabursally into rat ovaries with a 10 µl-syringe (Gaoge, China). The recombinant lentivirus of small interference RNA targeting LncRNA SNHG12 (si- LncRNA SNHG12) and the control lentivirus were prepared and titered to 10^9^ TU/mL (transfection unit). The needle was inserted slowly and held in place for 5 min. Each ovary was injected twice at different sites with 10 µl each time continuously for 7 days. The rats of the NC group were injected with si-RNA-NC as the negative control.

#### Sample collection

All rats were weighed once a week until the day of the virus injection. Two hours after the last dose, the body weight was measured, *n* = 5 in each group. All rats were euthanized by CO_2_ inhalation and fixed on the platform, and the ovarian tissue was removed and weighed. Blood and ovarian samples were then obtained from all rats.

#### Enzyme-linked immunosorbent assay (ELISA) measurement

The levels of follicle-stimulating hormone (FSH) (Rat, MM-0566R1), luteinizing hormone (LH) (Rat, MM-0624R1), estradiol (E_2_) (Rat, MM-0575R1), progesterone (P) (Rat, MM-0551R1), and testosterone (T) (Rat, MM-0577R1) in serum were measured using ELISA kits (Meimian, Jiangsu, China) following the manufacturer’s instructions, *n* = 5 in each group.

#### Hematoxylin-eosin (H&E) staining

The ovarian tissues which were were fixed with 4% paraformaldehyde, dehydrated using gradient ethanol and xylene, and then immersed in wax. Tissues were cut into slices at 5 μm and affixed to anti-peeling slides. The slices were baked at 60 °C for 12 h, dewaxed, hydrated with xylene and gradient ethanol, and stained with hematoxylin and eosin Stain (Servicebio, G1003). Finally, ethanol with low to high concentrations was added for dehydration. Vitrification was performed using xylene, and the slices were sealed. Finally, the sections were observed under a microscope (Nikon Eclipse E100).

#### Quantitative real time-PCR (qRT-PCR)

Pure ovarian tissue RNA was obtained by TRIzol (Sangon Biotech, B511311) extraction and transcribed into cDNA using a reverse transcription kit (Jiangsu Cowin Biotech CW2569). Primers (designed by Basic Local Alignment Search Tool (BLAST)), DEPC, cDNA, and SYBR Green (RR820A; Takara) were used to prepare the corresponding amplification products in the PCR instrument. The primer sequences used are listed in Table 1. The housekeeping gene were Human U6 and Rat U6. Fold changes in mRNA were calculated using the 2^−ΔΔCT^ method, *n* = 3 in each group.

**Table 1:** Primer sequences

Gene	Forward Primer	Reverse Primer
Human LncRNA SNHG12	TACAGAGATCCCGGCGTACT	CAACCAGGTCCCCTGCATTT
Human miR-129	GGATCTTTTTGCGGTCTGGG	ATACTTTTTGGGGTAAGGGCTTC
Human miR-125b	GTCCCTGAGACCCTAACTTGTG	CGACTCGCAGCTCCCAAGAG
Human U6	TCTGCTCCTATCCCAATTACCTG	ACTCCCGGATCTCTTCTAAGTTG
Rat LncRNA SNHG12	ACCGGATTTTTCCGTCTGGT	TCTGGTCTCCCTCCTCACAA
Rat miR-129	TGGGTCTTTTTGCGGTCTGG	AGATACTTTTTGGGGTAAGGGCT
Rat miR-125b	CCCCTCAGTCCCTGAGACC	AGCTCCCAAGAGCCTAACCC
Rat U6	GTGCAACAGCAGACCAGACT	TGCGCGAGGAATGGAAAGG

#### Western blot

Following the lysis of total protein of ovarian tissue, the BCA method was used to determine the protein concentration. After the loading buffer was applied, the boiling protein was denatured. Total protein was separated by electrophoresis, and the corresponding proteins were transferred to a membrane. The non-specific antigen was blocked with 5% milk, and the membrane containing the protein was incubated with the target antibodies (Affinity, USA): against Cyclin D1 (AF0931), CDK2 (AF6237), Bax (AF0120), Bcl-2 (AF6139), and the housekeeping control GAPDH (AF7021). After incubation at 4 °C for 14–16 h, unbound antibodies were washed and the membrane was additionally incubated with secondary antibodies. The unbound antibodies were washed again and captured using an ECL luminescence imager, *n* = 3 in each group.

### In vitro cell culture and treatment

Human granulosa tumor cell lines (KGN, COV434, and SVOG) and normal ovarian surface epithelial cells IOSE80 were purchased from the Shanghai Institutes for Biological Sciences, China. cell lines were cultured in DMEM supplemented with 100 IU/mL streptomycin, 100 IU/mL penicillin, and 10% fetal bovine serum (GIBCO, Carlsbad, CA,)/F12 medium (GIBCO, Carlsbad, CA, USA) and incubated in an incubator at 37 °C with 5% CO_2_ and. Different concentrations of insulin (5, 10, 25, and 30 ng/mL) were used to stimulate SVOG, COV434, and KGN cells to induce PCOS. Based on the effects of insulin on GCs, the groups were further divided into insulin, insulin + siRNA-NC, and insulin + siRNA-lncRNA SNHG12 groups.

#### CCK-8 assay

COV434 and KGN cell suspensions were seeded in 96-well plates and incubated for 24 h. Each well was filled with 10 µL of the CCK-8 assay (MCE, HY-K0301) solution and incubated for 24, 48, 72, and 96 h. Microplate readers were used to measure absorbance at 450 nm, *n* = 5 in each group.

#### 5-Ethynyl-2’-deoxyuridine (EdU) detection

KGN cells were cultured in 12-well plates with 60–70% cell density. The Cells were exposed to EdU (Beyotime, C0078s) for 4 h at 37 °C after transfection for 48 h. The samples were fixed in 95% ethanol for 15 min at room temperature. Hoechst 33,342 solution was then applied to each well and incubated for 2 min under light, and the results were evaluated by fluorescence microscopy (Ts2-FC, Nikon, Tokyo, Japan), *n* = 3 in each group.

#### Flow cytometry (FCM)

KGN cells (1.2 × 10^6^ per well in 6-well plates) were collected after 24 h of treatment according to the groups and washed with PBS. The supernatant was discarded by centrifugation, and 100 µL of binding buffer was added. Then, 10 µL of PI and 5 µL of Annexin V-FITC were added and thoroughly mixed. The reaction was performed in the dark for 15 min. Then, 400 µL of binding buffer was added, and the apoptosis rate was detected by FCM within 1 h, *n* = 3 in each group.

#### Quantitative real time-PCR (qRT-PCR)

Pure COV434 and KGN cell RNA was obtained by TRIzol (Sangon Biotech, B511311) extraction and transcribed into cDNA using a reverse transcription kit (Jiangsu Cowin Biotech CW2569). Primers, DEPC, cDNA, and SYBR Green (RR820A; Takara) were used to prepare the corresponding amplification products in the PCR instrument. The primer sequences used are listed in Table 1. The housekeeping gene were Human U6 and Rat U6. Fold changes in mRNA were calculated using the 2^−ΔΔCT^ method, *n* = 3 in each group.

#### Western blot

Following the lysis of total protein of COV434 and KGN cells, the BCA method was used to determine the protein concentration. After the loading buffer was applied, the boiling protein was denatured. Total protein was separated by electrophoresis, and the corresponding proteins were transferred to a membrane. The non-specific antigen was blocked with 5% milk, and the membrane containing the protein was incubated with the target antibodies (Affinity, USA): against Cyclin D1 (AF0931), CDK2 (AF6237), Bax (AF0120), Bcl-2 (AF6139), and the housekeeping control GAPDH (AF7021). After incubation at 4 °C for 14–16 h, unbound antibodies were washed and the membrane was additionally incubated with secondary antibodies. The unbound antibodies were washed again and captured using an ECL luminescence imager, *n* = 3 in each group.

#### Dual-luciferase reporter gene assay

The luciferase reporter vector was constructed. Accordingly, the luciferase was linked to the lncRNA SNHG12 gene and then transfected into cells for detection. 24 h post-transfection, luciferase activities were analyzed in KGN cells using the dual-luciferase reporter assay (Solarbio, D0010-100T), *n* = 3 in each group.

#### RNA binding protein immunoprecipitation (RIP) assay

The Pierce RNA 3’-end Magna RIP™ RNA-Binding Protein Immunoprecipitation Kit (Sigma Aldrich, 17–700) was used to label the RNAs with biotin. Next, different groups of RNAs were cultured with cell lysates. Subsequently, the magnetic beads were added to each binding reaction at room temperature and washed. Finally, qRT-PCR was used to test the expression of miR-129 and miR‐125b, *n* = 5 in each group.

### Statistical analysis

Statistical analysis was performed using the SPSS software (version 16.0, IBM, USA). When the measurement data in multiple groups were in accordance with a normal distribution and homogeneity of variance test, one-way ANOVA was used, and the Tukey test was used for further pairwise comparison between groups. Dunnett’s T3 test or an independent sample t-test was used if the distribution was normal, but the variance was not uniform. If it did not conform to the normal distribution, the Kruskal-Wallis H test was used. The significance level was set at *P* < 0.05. The data are shown as mean ± standard deviation.

## Results

### The injection of DHEA in rats influenced gonadal hormone, pathological changes, and the expressions of lncRNA SNHG12, miR-129, and miR-125b

**Fig. 1:**
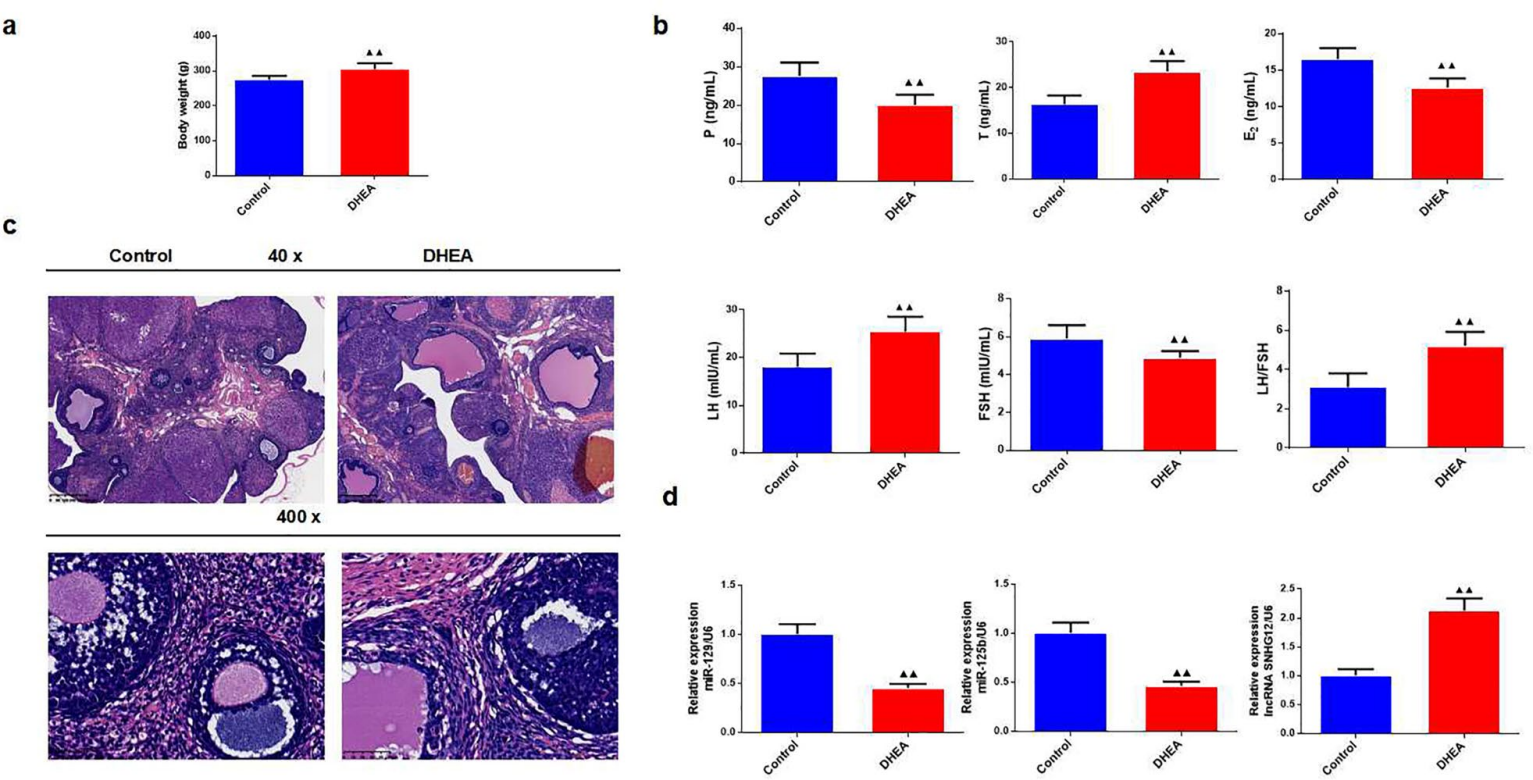
The injection of DHEA induced polycystic ovary syndrome (PCOS) model in rats. **a **Body weight of rats in each group, *n* = 5 in each group; **b **Enzyme-linked immunosorbent assay (ELISA) was used for testing testosterone (T), estradiol (E_2_), progesterone(P), luteinizing hormone (LH), and follicle-stimulating hormone (FSH) levels of the rats in each group, and the ratio of LH/FSH was calculated, *n* = 5 in each group; **c **The histomorphology of ovary tissues in rats was observed by hematoxylin-eosin staining (magnification ×40, 400); **d **Quantitative real-time PCR (qRT-PCR) was used for testing lncRNA SNHG12, miR-129, and miR-125b mRNA expression in rats in each group, *n* = 3 in each group; ^▲^*P* < 0.05, ^▲▲^*P* < 0.01 vs. control group

We administered DHEA injections to induce PCOS in SD rats. After DHEA modeling, the weight of the rats increased (Fig. 1a, *P* < 0.01). FSH, E_2_, P, T, and LH levels were measured using ELISA kits. After the injection of DHEA, the serum levels of T and LH were elevated, whereas E_2_, P, and FSH levels were evidently weakened, and the ratio of LH/FSH was increased (Fig. 1b, *P* < 0.01).

No pathological changes were observed in the ovarian tissues of the control group. Compared with the control group, the ovarian tissue of the DHEA group was more cystic-dilated, and the number of GCs layers decreased significantly (Fig. 1c).

After injection of DHEA, we found that lncRNA SNHG12 intensified; however, miR-129 and miR-125b were restrained (Fig. 1d, *P* < 0.01).

### LncRNA SNHG12 influenced body weight, ovary weight, gonadal hormone, miR-129, miR-125b expressions, and pathological changes in PCOS rats

**Fig. 2:**
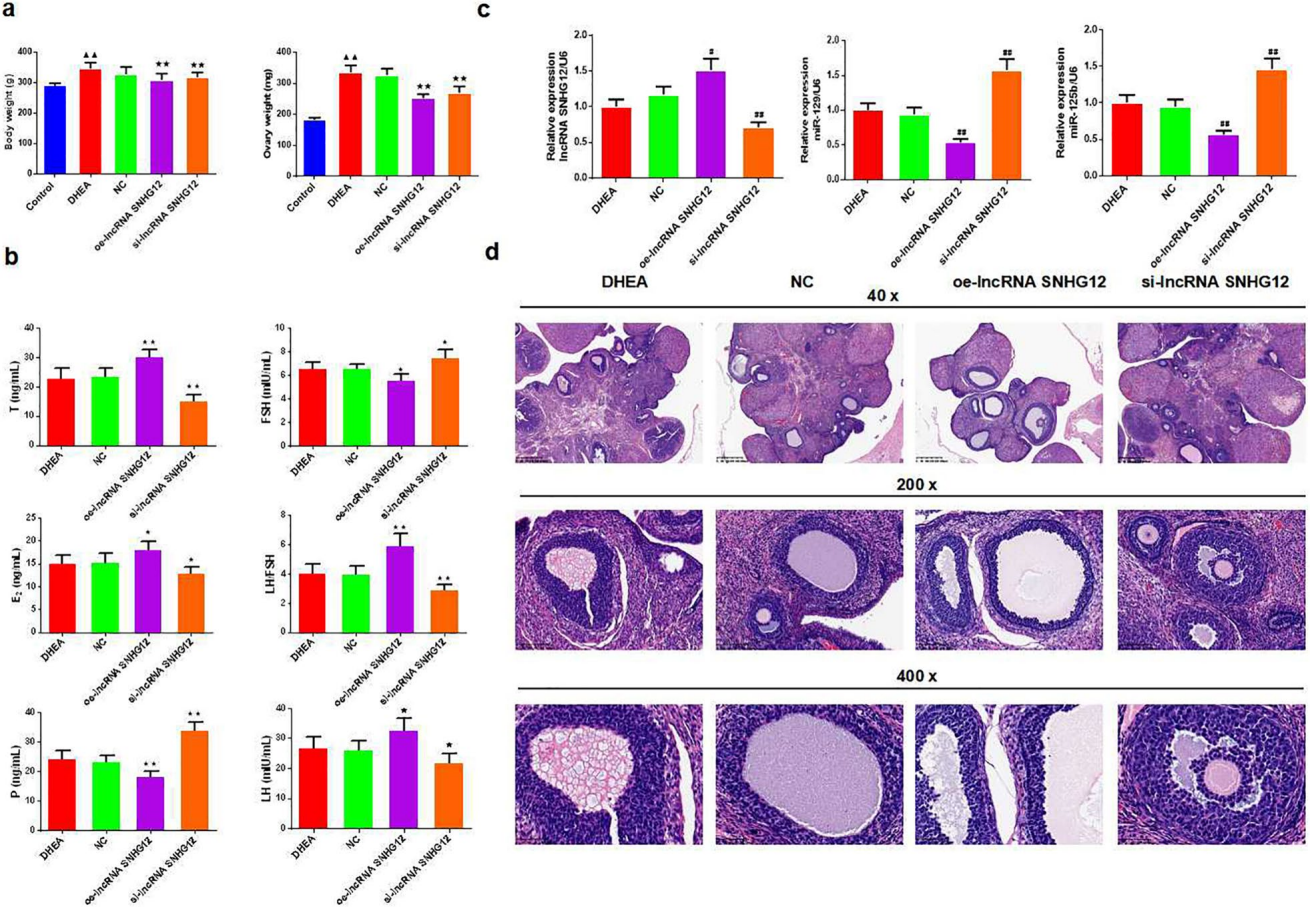
Overexpressing and silencing the expression of lncRNA SNHG12 influenced the body weight, ovary weight, gonadal hormone, miR-129, miR-125b expressions, and pathological changes in PCOS rats. **a **Body weight and ovary weight of rats in each group, *n* = 5 in each group; **b **ELISA was used for testing the T, E_2_, P, LH and FSH levels of the rats in each group, the ratio of LH/FSH was calculated, *n* = 5 in each group; **c **qRT-PCR was used for testing lncRNA SNHG12, miR-129, and miR-125b mRNA expressions of rats in each group, *n* = 3 in each group; **d **The histomorphology of ovary tissues in rats was observed by hematoxylin-eosin staining (magnification × 40, 200, 400); ^▲^*P* < 0.05, ^▲▲^*P* < 0.01 vs. control group; ^★^*P* < 0.05, ^★★^*P* < 0.01 vs. DHEA group; *P* < 0.05, ^##^*P* < 0.01 vs. NC group

Based on DHEA injection, we established oe-lncRNA SNHG12, si-lncRNA SNHG12, or NC groups. The results showed that the body weight and ovary weight of the oe-lncRNA SNHG12 and si-lncRNA SNHG12 groups were higher than those of the DHEA group (Fig. 2a, *P* < 0.01).

Furthermore, T, E_2,_ and LH were expressed at higher levels in oe-lncRNA SNHG12 with lower FSH and P expression, and the ratio of LH/FSH increased compared to that in DHEA rats (Fig. 2b; *P* < 0.01). The oe-lncRNA SNHG12 upregulated the relative expression of SNHG12 and downregulated the expression of miR-129 and miR-125b in PCOS rats compared to the NC group (Fig. 2c, *P* < 0.01). si-lncRNA SNHG12 demonstrated the opposite effect (Fig. 2b, c, *P* < 0.01).

Significant cystic dilatation was observed in the ovarian tissue sections, and the number of granulosa cell layers was reduced in the DHEA and NC groups. However, the ovarian tissue of the oe-lncRNA SNHG12 group worsened and cystic dilatation was severe, while the pathological manifestations of the si-lncRNA SNHG12 group were alleviated (Fig. 2d).

### LncRNA SNHG12 expressed the highest in KGN cells

**Fig. 3:**
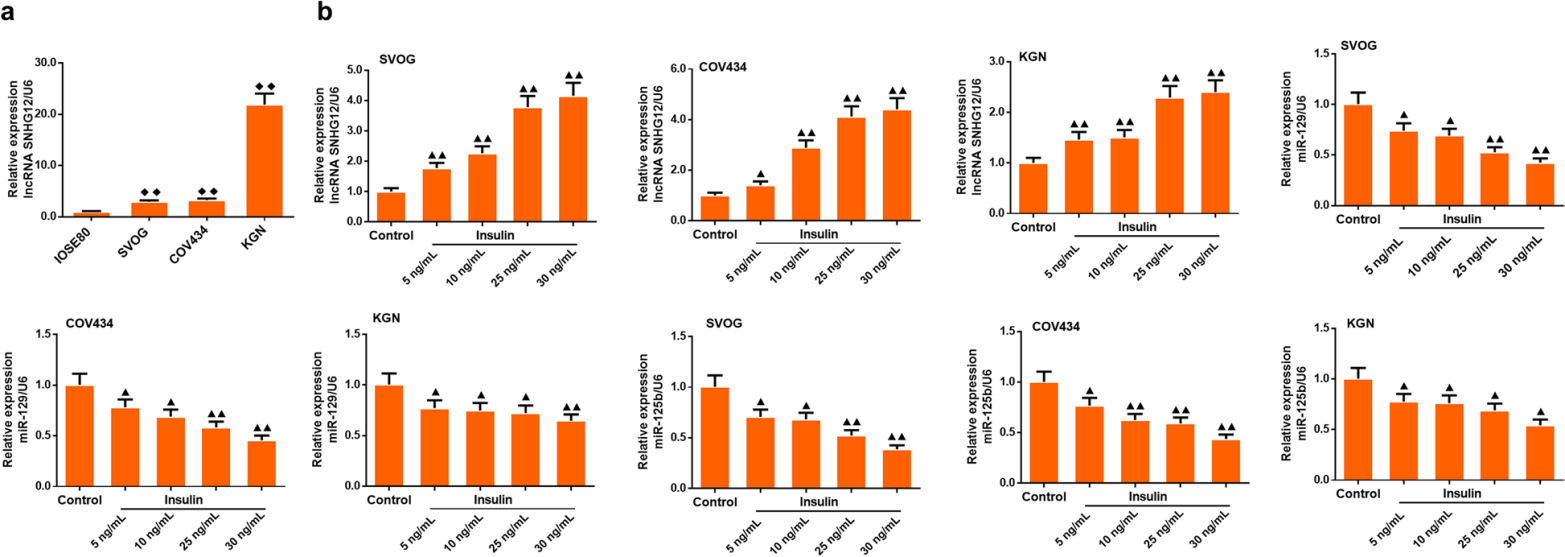
The administration of insulin to IOSE80, SVOG, COV434, and KGN cells to induce PCOS model in vitro*. ***a **qRT-PCR was used for testing lncRNA SNHG12 level in each group of cells; ^♦^*P* < 0.05, ^♦♦^*P* < 0.01 vs. IOSE80 cell group, *n* = 3 in each group; **b **qRT-PCR was used for testing lncRNA SNHG12, miR-129, and miR-125b expressions under the different dosages of insulin in SVOG, COV434 and KGN cells; *n* = 3 in each group; ^▲^*P* < 0.05, ^▲▲^*P* < 0.01 vs. control group

Cell experiments were performed on IOSE80, SVOG, COV434, and KGN cells. qRT-PCR showed that compared with the IOSE80 group, the expression of lncRNA SNHG12 was significantly upregulated in SVOG, COV434, and KGN cells, and lncRNA SNHG12 was the highest in KGN cells (Fig. 3a, *P* < 0.01).

### The expressions of lncRNA SNHG12, miR-129, and miR-125b after the intervention with different dosages of insulin in SVOG, COV434, and KGN cells

In vitro, we simulated SVOG, COV434, and KGN cells with insulin to mimic PCOS in humans. The results demonstrated that the expression of lncRNA SNHG12 in SVOG, COV434, and KGN cells was increased by insulin treatment in a dose-dependent manner, whereas the expression of miR-129 and miR-125b was decreased by insulin treatment in a dose-dependent manner in these cells compared to the control group (Fig. 3b, *P* < 0.05 or *P* < 0.01).

### Silencing lncRNA SNHG12 restrained cell viability, and attenuated cell proliferation, but strengthened apoptosis in KGN cells

**Fig. 4:**
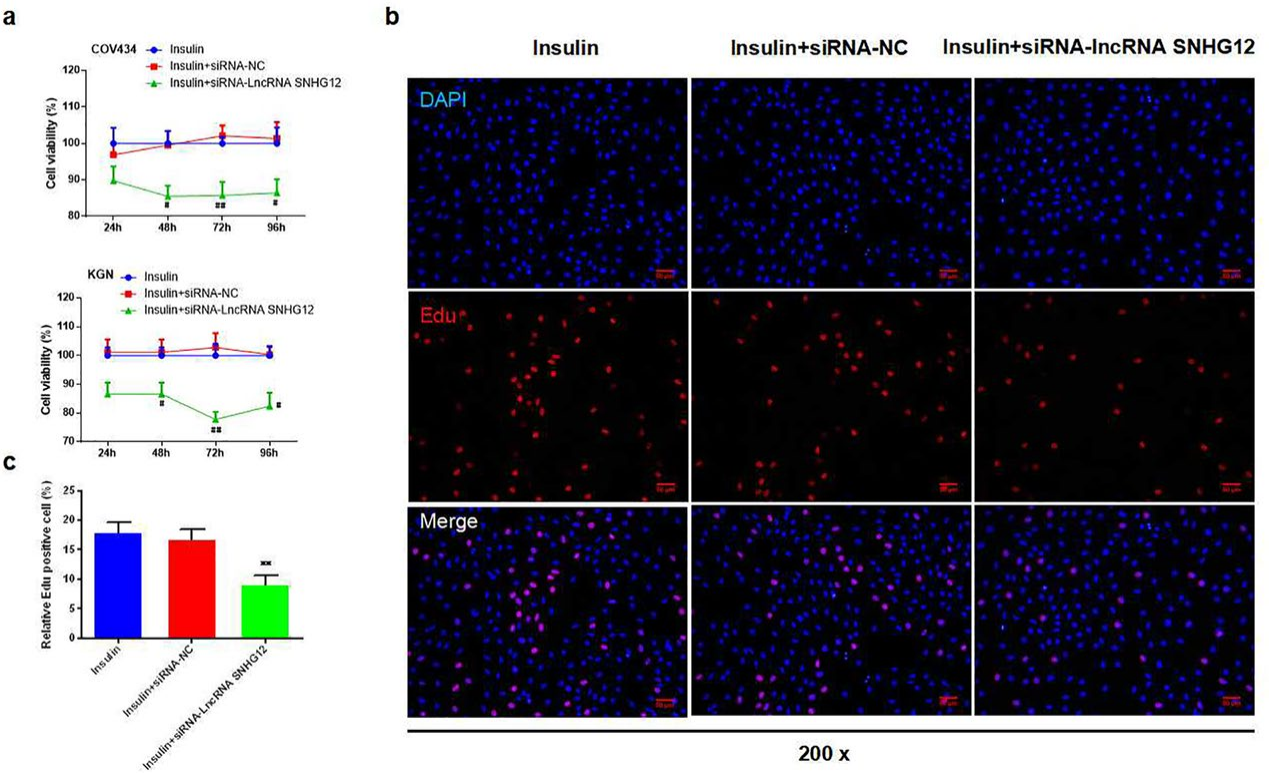
The inhibition effect of silencing lncRNA SNHG12 on the viability and proliferation of granule cells (GCs). **a **CCK-8 assay was used to test cell viability at 24, 48, 72, and 96 h in COV434 and KGN cells in each group, *n* = 5 in each group; **b **Immunofluorescence tested the DAPI and EdU co-labeled antibodies in KGN cells in each group; **c **The percentage of EdU-positive cells in KGN cells; *n* = 3 in each group; ^#^*P* < 0.05, ^##^*P* < 0.01 vs. insulin + siRNA-NC group

**Fig. 5:**
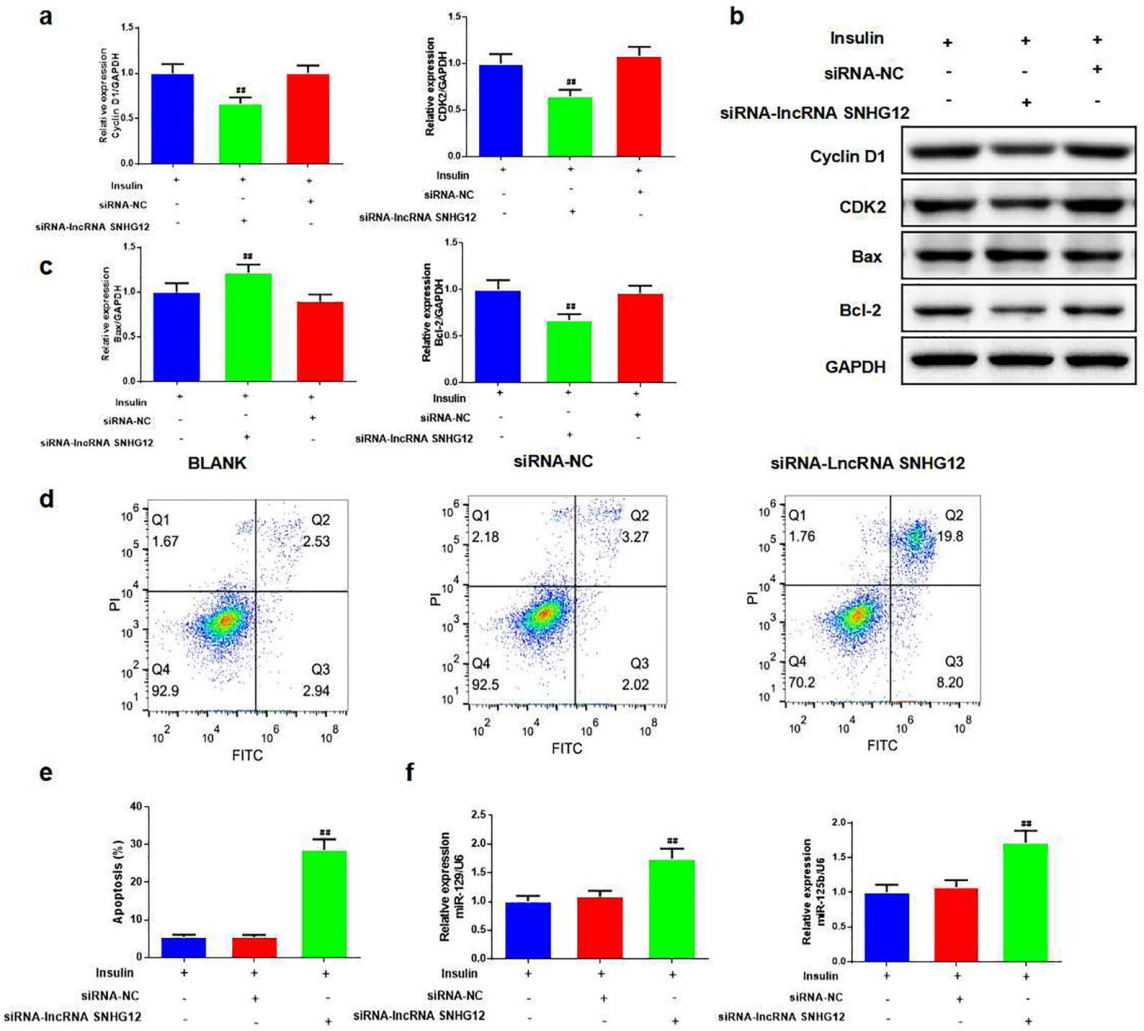
Silencing lncRNA SNHG12 strengthened apoptosis and the expression of miR-129, and miR-125b in KGN cell. **a**–**c **Expression levels of cyclin D1, CDK2, Bax, and Bcl-2 proteins were measured by western blotting, and the analysis in each group, *n* = 3 in each group; **d**,** e **Flow Cytometry (FCM) was used for testing the apoptosis in each group, and the analysis of the FCM with the cell apoptosis, *n* = 3 in each group; **f **qRT-PCR was used for testing the miR-129 and miR-125b expression after silencing lncRNA SNHG12 in each group; *n* = 3 in each group; ^#^*P* < 0.05, ^##^*P* < 0.01, vs. insulin + siRNA-NC group

Based on insulin treatment, the CCK-8 assay was used to test the viability of COV434 and KGN cells transfected with si-lncRNA SNHG12. The results showed that the cell viability of the siRNA-lncRNA SNHG12 group was decreased at 48 h, 72 h, and 96 h compared to the siRNA-NC group in both COV434 and KGN cells, especially at 72 h (Fig. 4a, *P* < 0.05, or *P* < 0.01). We then used immunofluorescence to stain EdU and observe cell proliferation in KGN cells (Fig. 4b). After silencing lncRNA SNHG12, the relative percentage of EdU-positive cells was evidently suppressed (Fig. 4c, *P* < 0.01) with a reduction in Cyclin D1, CDK2, and B cell lymphoma/leukemia-2 gen (Bcl-2) protein expression, whereas Bax expression was intensified compared to the siRNA-NC group (Fig. 5a–c, *P* < 0.01).

FCM results showed that silencing lncRNA SNHG1 promoted the cell apoptosis rate compared to the siRNA-NC group (Fig. 5d, e, *P* < 0.01).

### Inhibitions of miR-129 and miR-125 offset the effect of silencing lncRNA SNHG12 in KGN cells

**Fig. 6:**
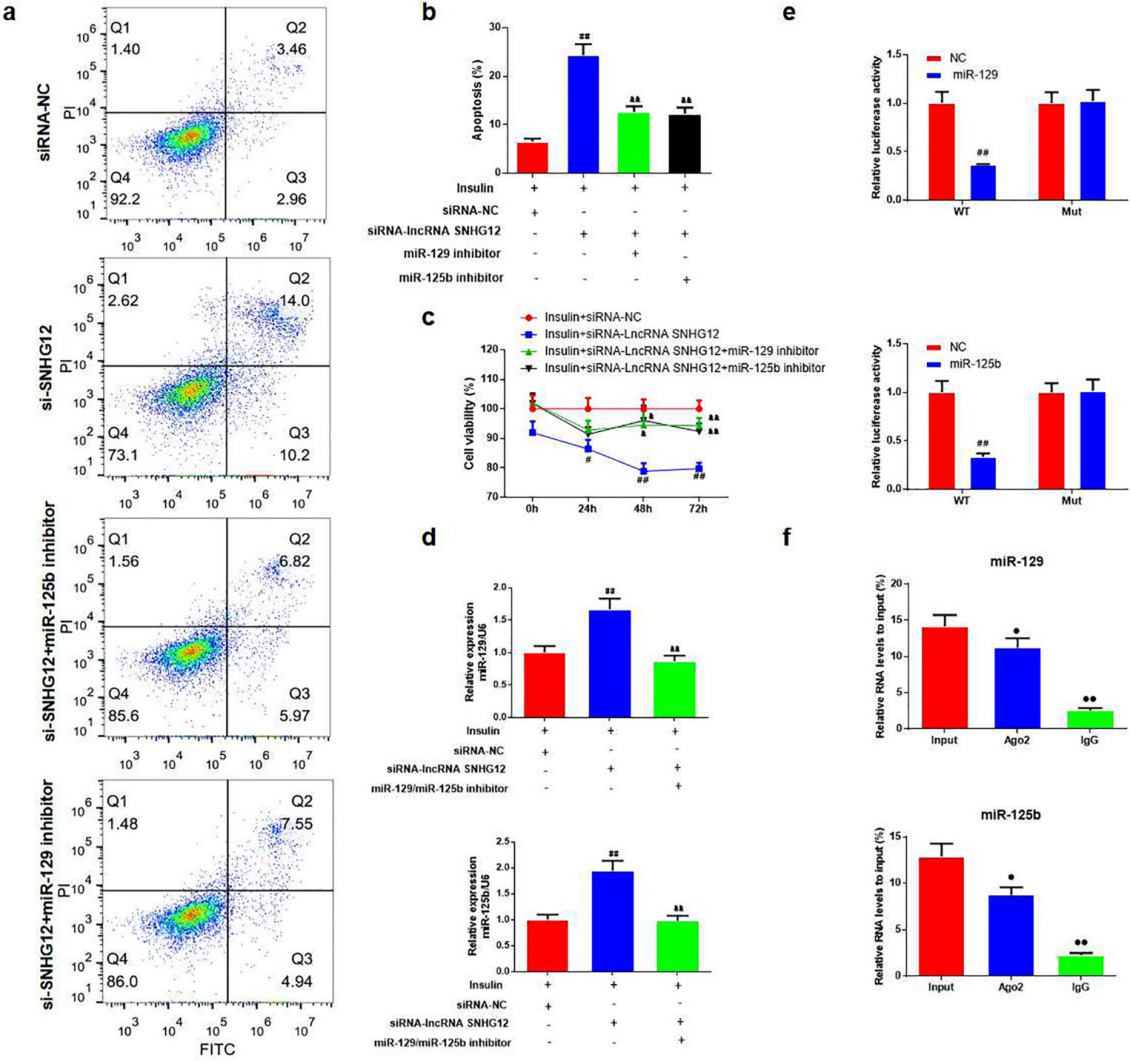
The effect of miR-129 and miR-125b in KGN cells with silencing lncRNA SNHG12. **a**,** b **FCM was used for testing the apoptosis in KGN cells in each group and the analysis of cell apoptosis, si- SNHG12: si-RNA-lncRNA SNHG12, *n* = 3 in each group; **c **CCK-8 assays were used to test cell viability at 24, 48, 72, and 96 h in KGN cells in each group, *n* = 5 in each group; **d **The levels of miR-129 and miR-125b in KGN cells with miR-129 and miR-125b inhibitor treatment were measured by qRT-PCR, *n* = 3 in each group; ^#^*P* < 0.05, ^##^*P* < 0.01 vs. insulin + siRNA-NC group; ^**&**^*P* < 0.05, ^**&&**^*P* < 0.01 vs. insulin + siRNA-lncRNA SNHG12 group; **e **The relative luciferase ratio in KGN cells co-transfected with SNHG12 WT luciferase vector and miR-129, miR-125b mimics, *n* = 3 in each group, ^#^*P* < 0.05, ^##^*P* < 0.01 vs. insulin + siRNA-NC group; **f **Anti-Ago2 RNA Binding Protein Immunoprecipitation (RIP) assay was used to pull down endogenous RNAs associated with Ago2; IgG served as the control. The levels of miR-129 and miR-125b were measured by qRT-PCR. *n* = 5 in each group, ^**•**^*P* < 0.05, ^**••**^*P* < 0.01 vs. the input group

The data showed that after silencing lncRNA SNHG12, miR-129, and miR-125b expression was higher than that in the siRNA-NC group (Fig. 5f, *P* < 0.01). In addition, we co-transfected miR-129 and miR-125b inhibitors into KGN cells with the silenced lncRNA SNHG12. Using FCM, we found that miR-129 and miR-125b inhibitors reduced cell apoptosis compared to the si-lncRNA SNHG12 group (Fig. 6a, b, *P* < 0.01). Cell viability after treatment with miR-129 and miR-125b inhibitors was enhanced compared to that of the si-lncRNA SNHG12 group (Fig. 6c, *P* < 0.01). The expression levels of miR-129 and miR-125b were undoubtedly reduced by the inhibitors in the si-lncRNA SNHG12 group (Fig. 6d, *P* < 0.01).

### Interactions between miR-129, miR-125b and lncRNA SNHG12

A dual-luciferase reporter gene was used to explore the interactions between miR-129, miR-125b, and SNHG12. The results showed that the luciferase ratio of miR-129 and miR-125b combined with the lncRNA SNHG12 3´-UTR was significantly lower than that of the lncRNA SNHG12-NC group. These results showed that miR-129 and miR-125b significantly reduced the relative luciferase activity of lncRNA SNHG12 (Fig. 6e, *P* < 0.01) but had no significant effect on lncRNA SNHG12-MUT. The RIP assay showed that compared to the input group, the expressions of miR- 129 and miR-125b in the Ago2 and IgG groups were significantly decreased (Fig. 6f, *P* < 0.05 or *P* < 0.01).

## Discussion

PCOS is the most prevalent endocrine problem in women of reproductive age and is frequently accompanied by hyperandrogenism, hyperinsulinism, and cytokine secretion dysfunction [16, 17]. Several studies have suggested that PCOS is associated with increased ovarian GCs apoptosis [18–21]. As a key regulator of human disease, an increasing number of studies have shown that lncRNAs are related to PCOS, which plays a role in GCs proliferation and apoptosis and participates in almost every link between the occurrence and development of PCOS [20, 22–24].

This study was performed to determine the role of the lncRNAs SNHG12, miR-129, and miR-125b in GCs of PCOS patients. First, we found a disorder in the pathological structure of the ovarian tissue and sex hormones in PCOS rats induced by DHEA. For instance, T and LH levels in the serum were elevated, whereas E_2_, P, and FSH levels were reduced. The LH/FSH ratio was also increased. Similar to the study of Liu [25], this study revealed that DHEA modeling induced PCOS, causing the disorder of sex hormones. In addition, overexpression of lncRNA SNHG12 in PCOS rats influenced sex hormones in the same way, whereas silencing of SNHG12 acted conversely, indicating that lncRNA SNHG12 influenced the balance of sex hormones in PCOS.

It has been reported that lncRNA SNHG12 increases cell growth and inhibits apoptosis in cancer cells [26]. Overexpression of EdU and Cyclin D1 contributes to uncontrolled cell proliferation and malignancy in PCOS [27]. CDK binds to cyclin to form heterodimers [28]. Overexpression of Bax could antagonize the protective effect of the Bcl-2, resulting in cell death [18]. Similar to previous reports, in our study, which was attributed to the silencing of the lncRNA SNHG12 in the insulin-induced PCOS model in KGN cells, cell viability and cell proliferation were attenuated with the reduction of EdU, Cyclin D1, and CDK proteins. In contrast, silencing of lncRNA SNHG12 promoted cell apoptosis. The cell apoptosis-related protein Bax was upregulated, whereas Bcl-2 was downregulated.

miRNAs play essential roles in several cellular processes. With the deepening of molecular biology research, an increasing number of experts believe that some miRNAs bind to the lncRNA SNHG12 and participate in the progression of diseases of the female reproductive system. A study showed that the lncRNA SNHG12 absorbing miRNA-129 speeds up the progression of ovarian cancer [13]. Another study indicated that SNHG12 plays a significant regulatory role in the progression of cervical cancer by modulating the miR-125b/STAT3 axis [14]. Zhu et al. [29] found that miR-129 affects endocrine disturbances, proliferation, and apoptosis of ovarian GCs in PCOS. The significant regulator miR-125b is related to apoptosis in a diverse range of cell types, both in normal and cancer cells. Yao [30] showed that miR-125b promoted apoptosis in GCs. In this study, the results demonstrated that the expression of lncRNA SNHG12 influenced cell apoptosis by reducing miR-129 and miR-125b in PCOS rats and cells. Based on bioinformatics prediction, miR-129 and miR-125b were predicted to be downstream of lncRNA SNHG12, which was further proven by dual-luciferase reporter assay and RIP in our study that miR-129 and miR-125b can bind to lncRNA SNHG12.

Recent studies have further expanded our understanding of the role of lncRNAs in PCOS. For instance, research by Li et al. [31] and Liu et al. [32] have highlighted the complex interplay between various lncRNAs and miRNAs, suggesting a broader regulatory network involved in PCOS pathogenesis beyond the scope of our current study. These findings underscore the potential for targeting multiple components of this network as a therapeutic strategy for PCOS.

In summary, our study has demonstrated that the injection of DHEA to induce a PCOS model in rats, coupled with the overexpression of lncRNA SNHG12, significantly impacts body and ovary weight, alters gonadal hormone levels, and induces pathological changes. These effects are mediated through the modulation of miR-129 and miR-125b expression, highlighting the intricate molecular interplay involved in PCOS pathogenesis. Conversely, silencing lncRNA SNHG12 in both rat models and KGN cells leads to decreased cell viability and proliferation, alongside an increase in apoptosis, underscoring the pivotal regulatory role of lncRNA SNHG12 in PCOS development.

Furthermore, our findings on the interaction between lncRNA SNHG12, miR-129, and miR-125b provide valuable insights into the molecular mechanisms by which lncRNA SNHG12 may promote cell proliferation and inhibit apoptosis in the context of PCOS. This interaction suggests a complex regulatory network that could offer new targets for therapeutic intervention.

The implications of our research are manifold. Firstly, it underscores the potential of lncRNA SNHG12 as a biomarker for diagnosing or monitoring the progression of PCOS. Secondly, the regulatory mechanisms uncovered in this study offer promising targets for novel therapeutic strategies aimed at modulating the expression of lncRNA SNHG12, miR-129, and miR-125b to mitigate the symptoms or halt the progression of PCOS.

However, our study is not without limitations. The in vivo experiments were conducted on a rat model, which, while informative, may not fully replicate the human condition. Additionally, the in vitro studies were limited to granulosa-like tumor cells, which may behave differently from normal granulosa cells found in human ovaries. Therefore, further research involving human subjects and a broader range of cell types is essential to validate our findings and explore their clinical applicability.

Future studies should aim to elucidate the detailed molecular pathways through which lncRNA SNHG12 influences PCOS development and progression. Investigating the potential interactions between lncRNA SNHG12 and other miRNAs or molecular targets could also provide deeper insights into the complex network of gene regulation in PCOS. Moreover, clinical trials are necessary to assess the feasibility, safety, and efficacy of targeting lncRNA SNHG12 for therapeutic purposes.

In conclusion, our study contributes to the growing body of evidence supporting the significant role of lncRNAs in the pathogenesis of PCOS. By shedding light on the regulatory functions of lncRNA SNHG12, this research paves the way for further experimental and clinical studies aimed at exploring innovative diagnostic and therapeutic approaches for PCOS, ultimately improving the quality of life for affected individuals.

### Supplementary Information

**Supplementary Material 1.**

## Data Availability

No datasets were generated or analysed during the current study.

## Abbreviations

PCOS: Polycystic ovary syndrome
IR: Insulin resistance
GCs: Granulosa cells
lncRNA: Long non-coding RNA
miRNAs: Micro-RNAs
SPF: Specific pathogen-free
h: Hour
DHEA: Dehydroepiandrosterone
ELISA: Enzyme-linked immunosorbent assay
FSH: Follicle-stimulating hormone
LH: Luteinizing hormone
E_2_: Estradiol
P: Progesterone
T: Testosterone
H&E: Hematoxylin-eosin
qRT-PCR: Quantitative real time-PCR
EdU: 5-Ethynyl-2’-deoxyuridine
FCM: Flow cytometry
RIP: RNA Binding Protein Immunoprecipitation
