# Supplementary figures and images for: LncRNA SNHG12 promotes cell proliferation and inhibits apoptosis of granulosa cells in polycystic ovarian syndrome by sponging miR-129 and miR-125b
Source: J Ovarian Res. 2024 Apr 2;17:72. doi: 10.1186/s13048-024-01392-6 (PMC10986130; doi:10.1186/s13048-024-01392-6)

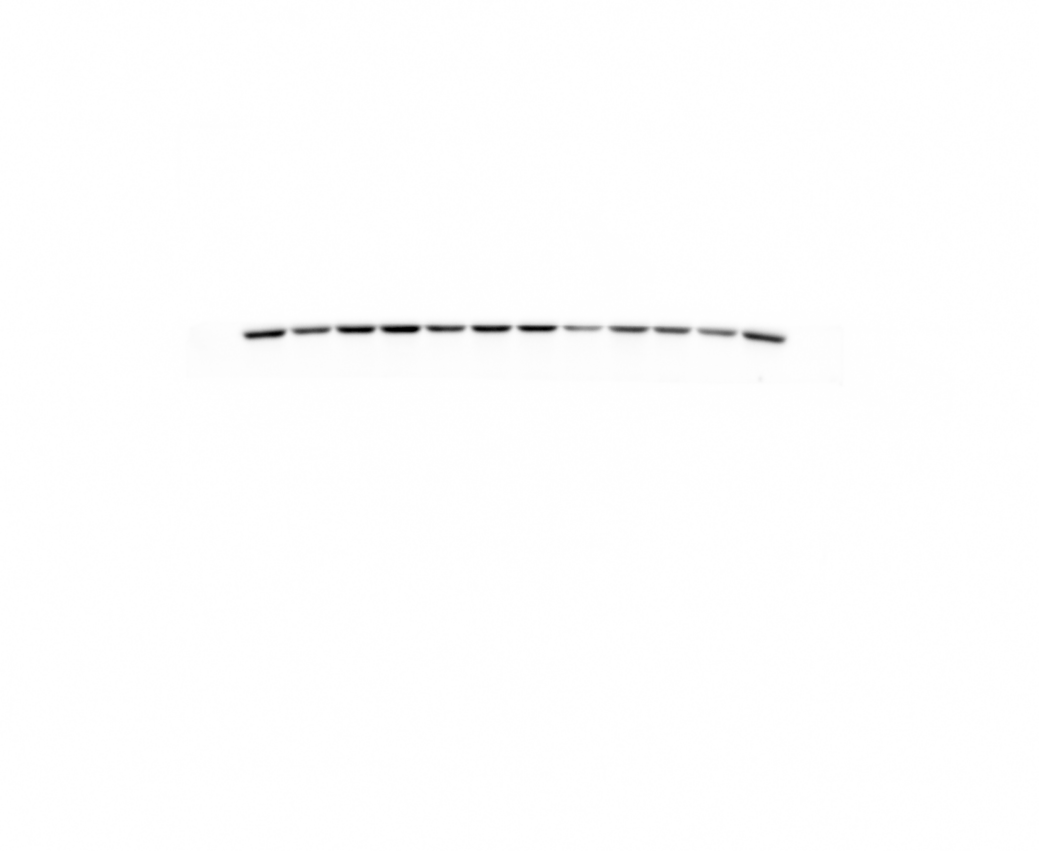


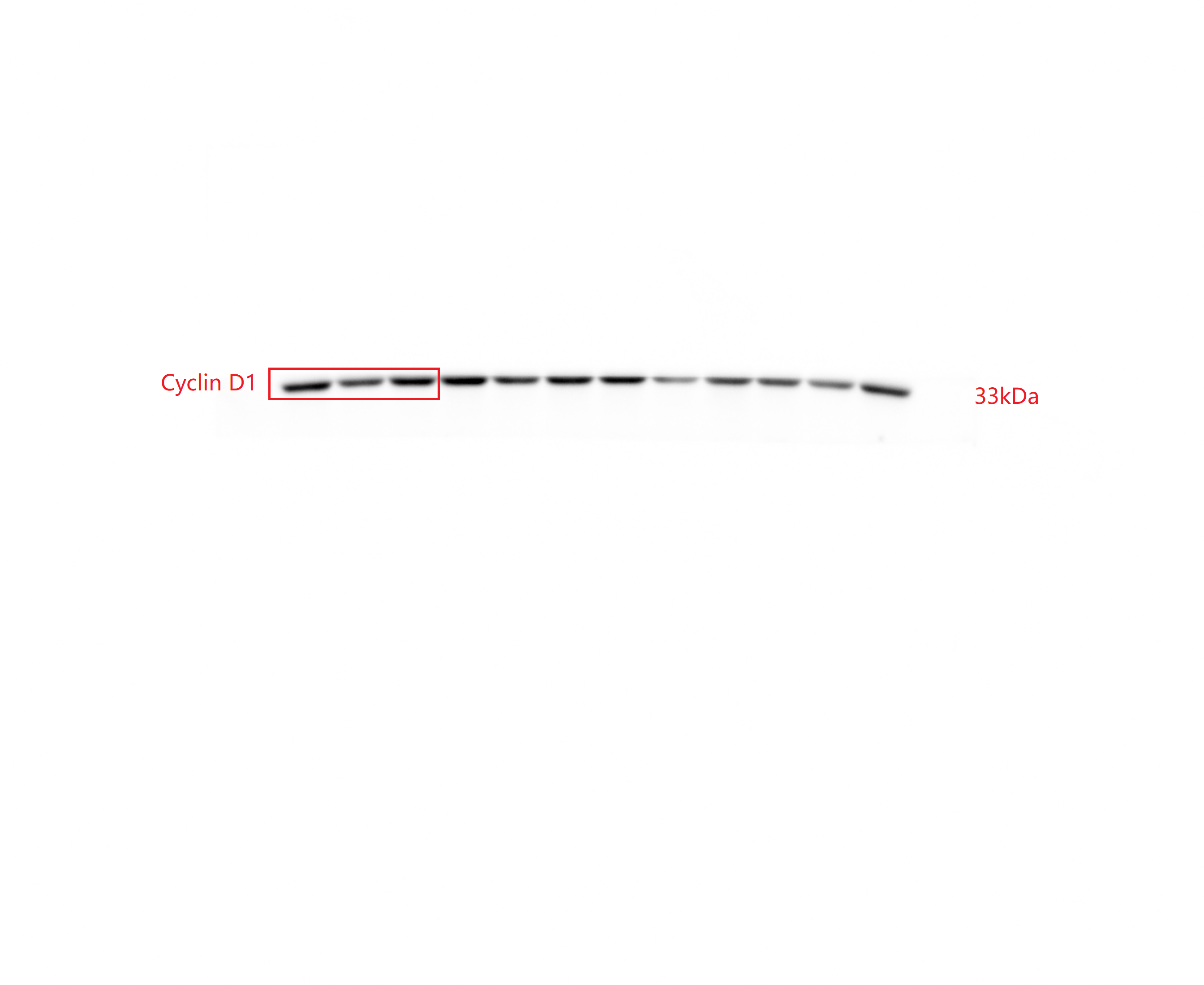


Figure 5 (Cyclin D1)


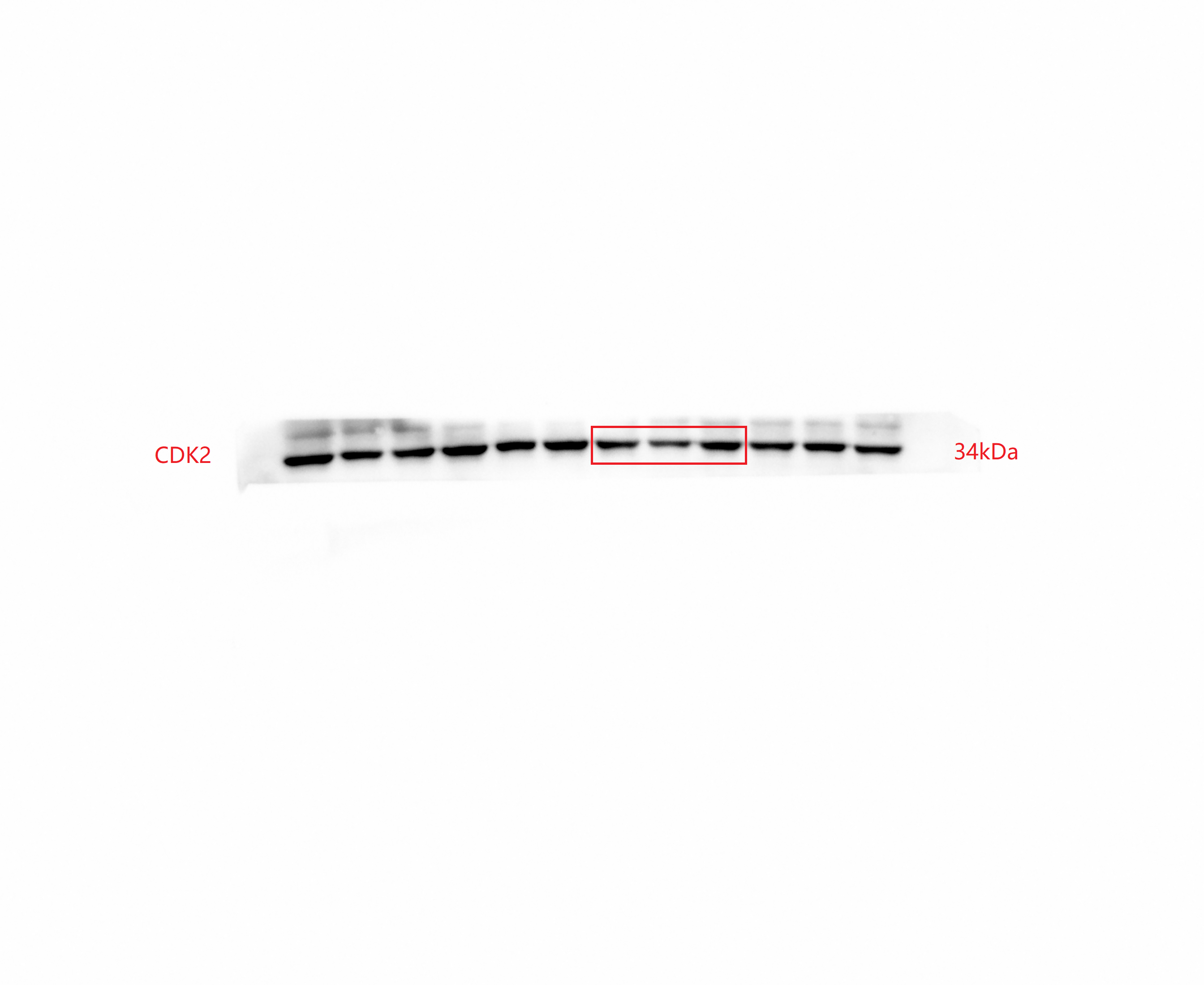


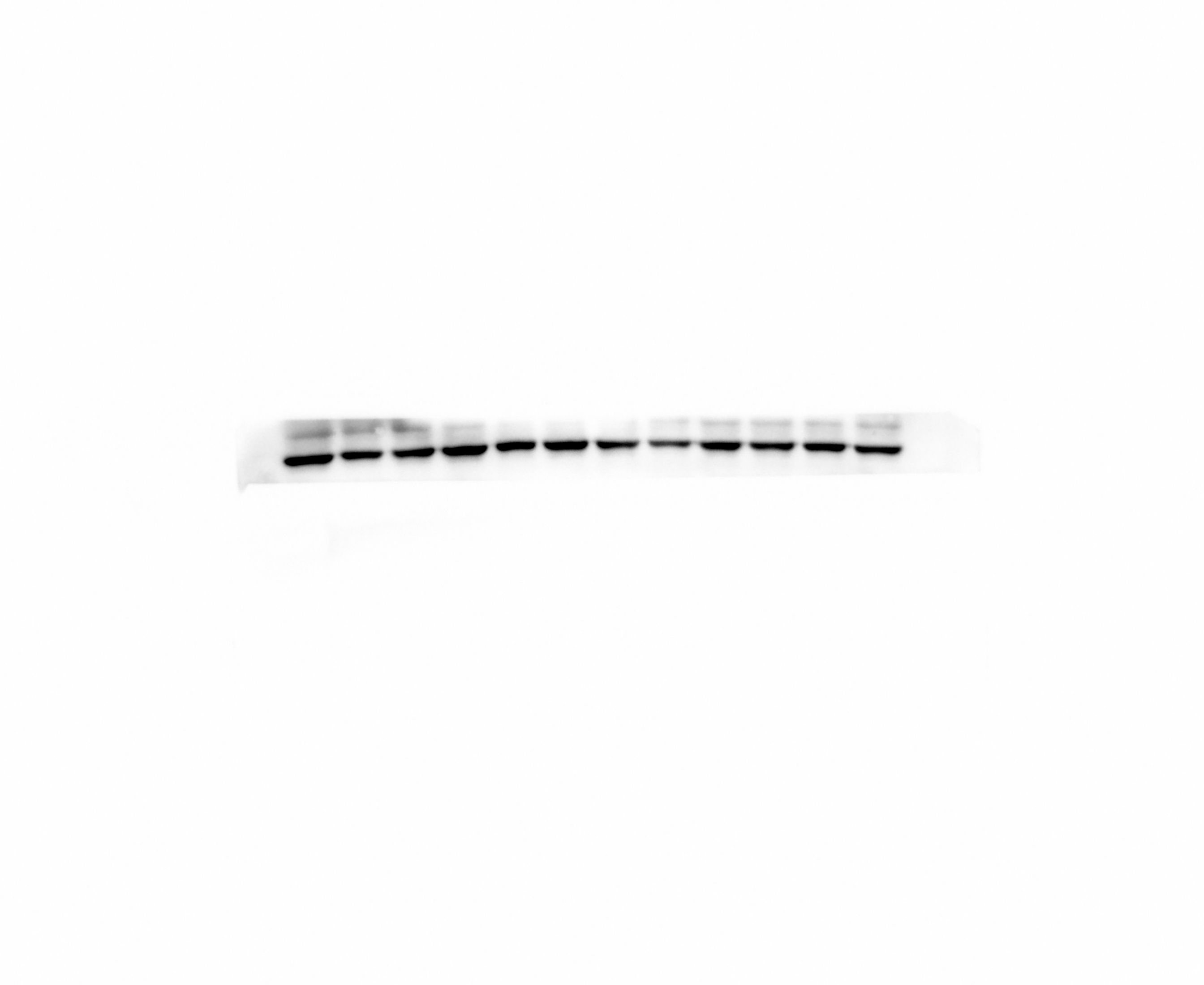


Figure 5 (CDK2)


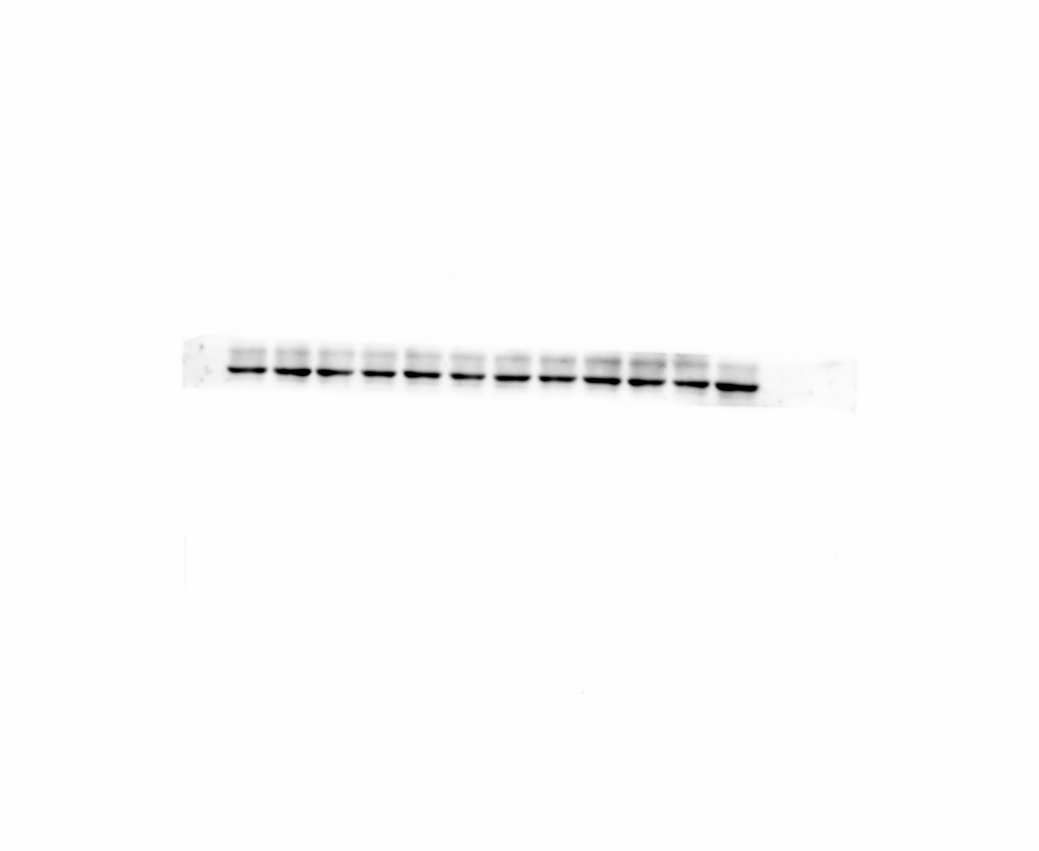


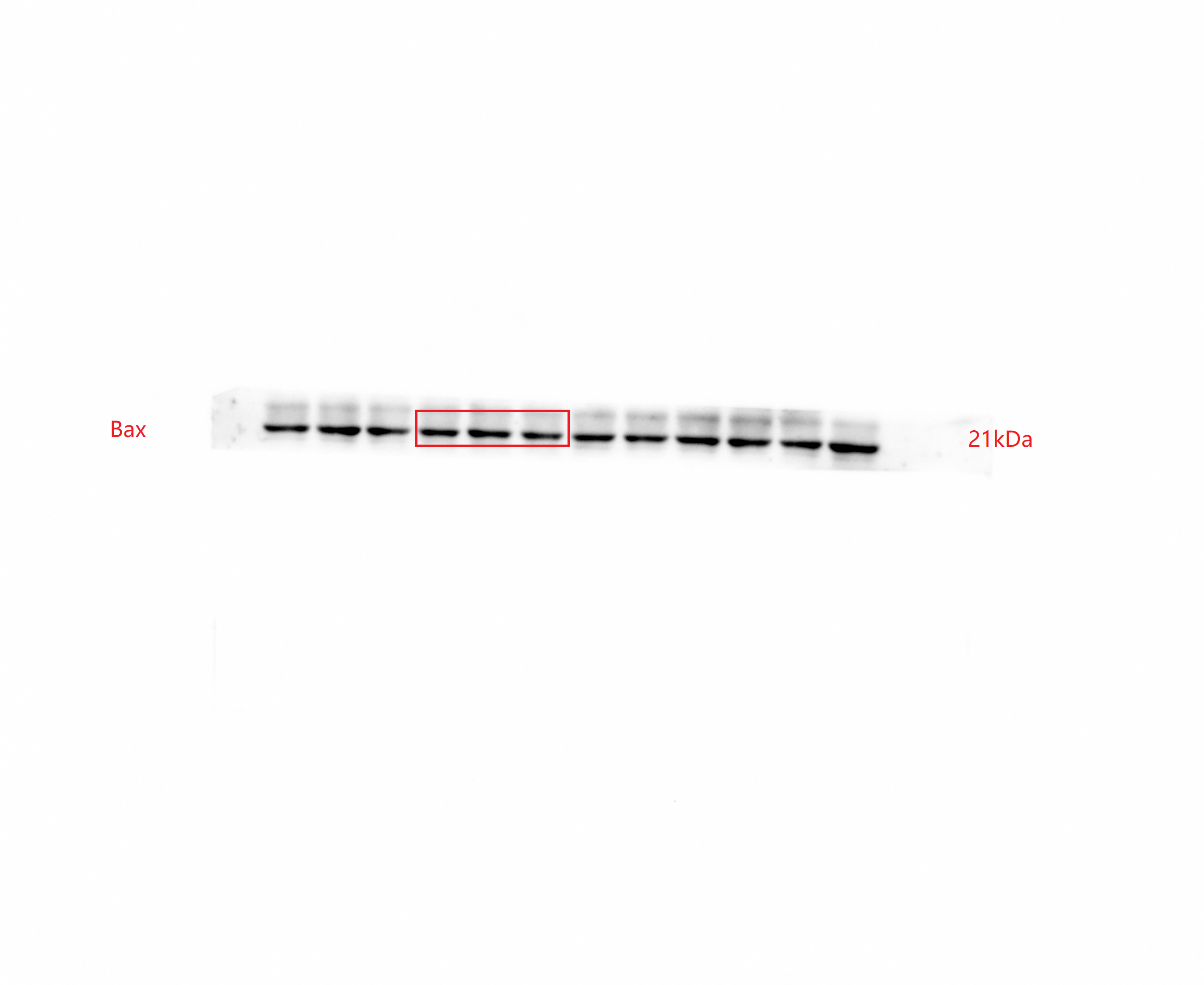


Figure 5 (Bax)


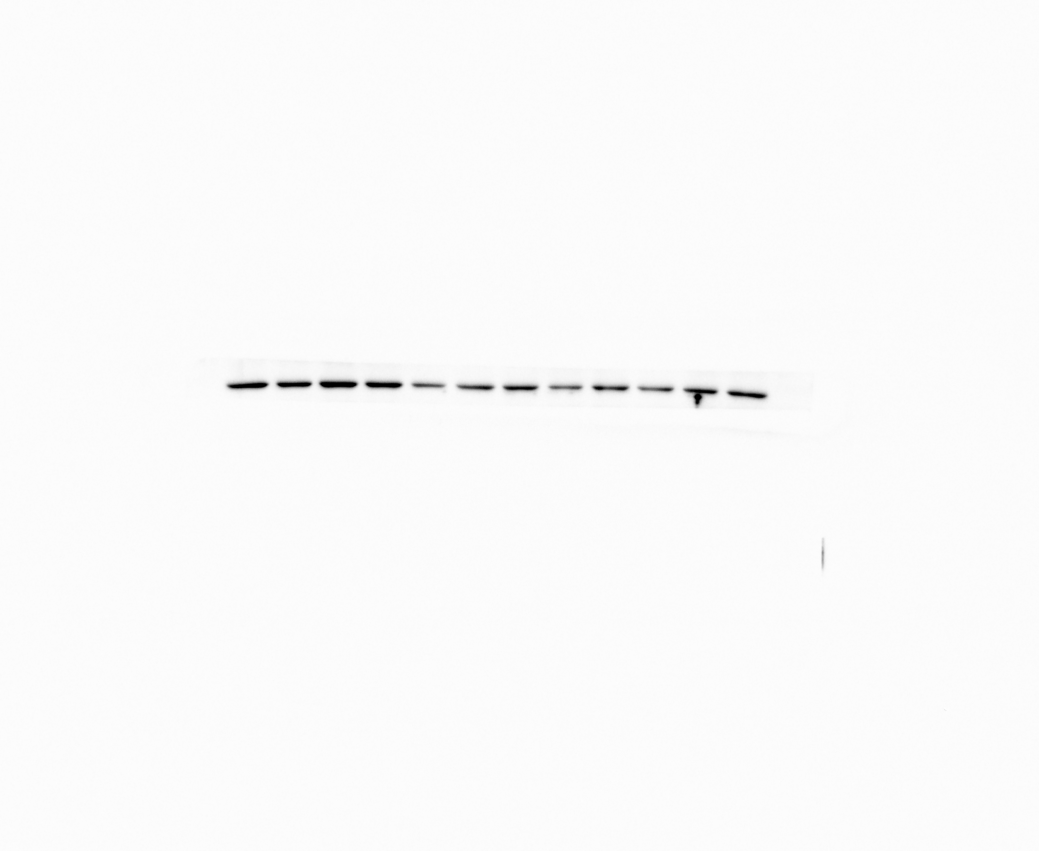


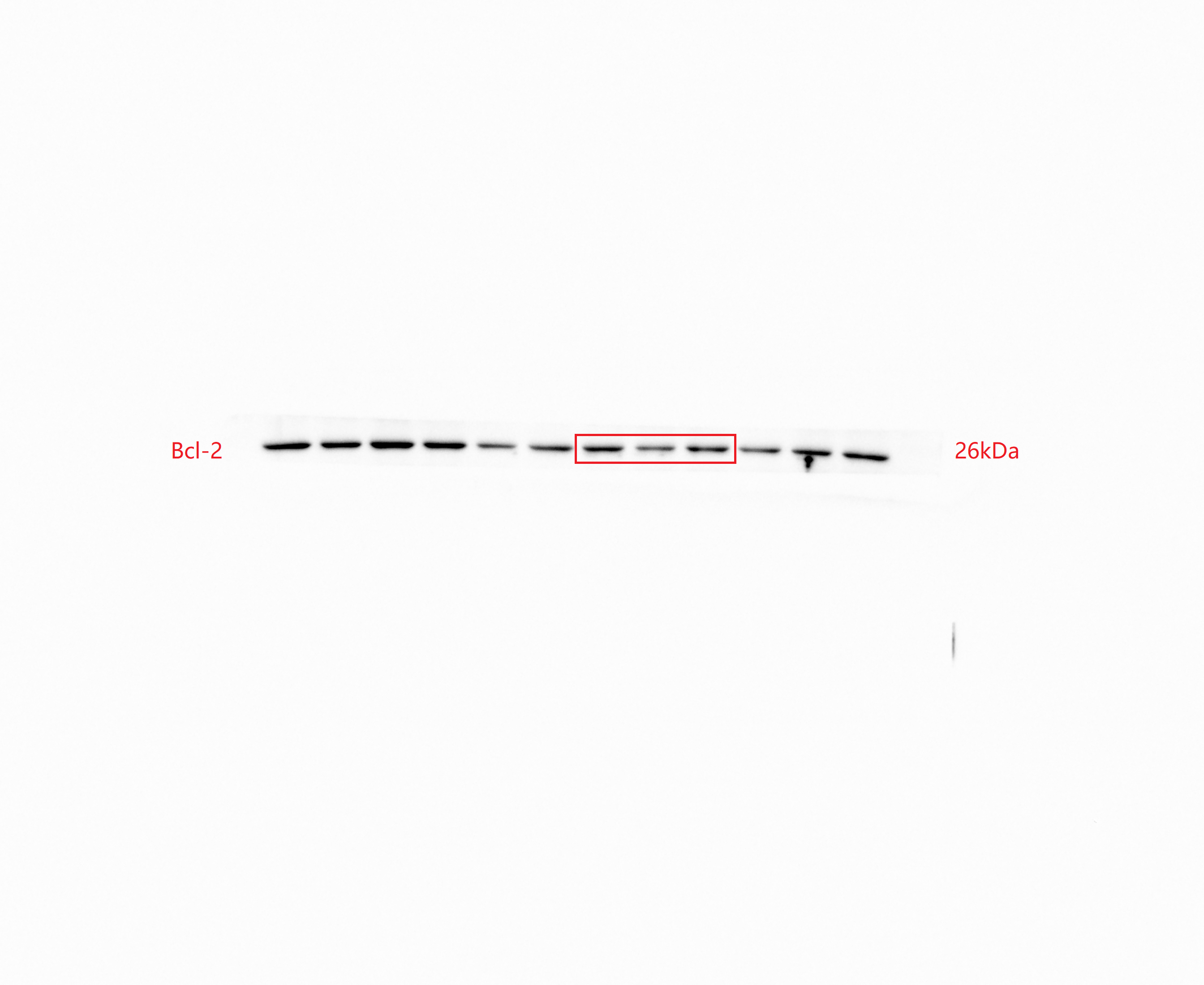


Figure 5 (Bcl-2)


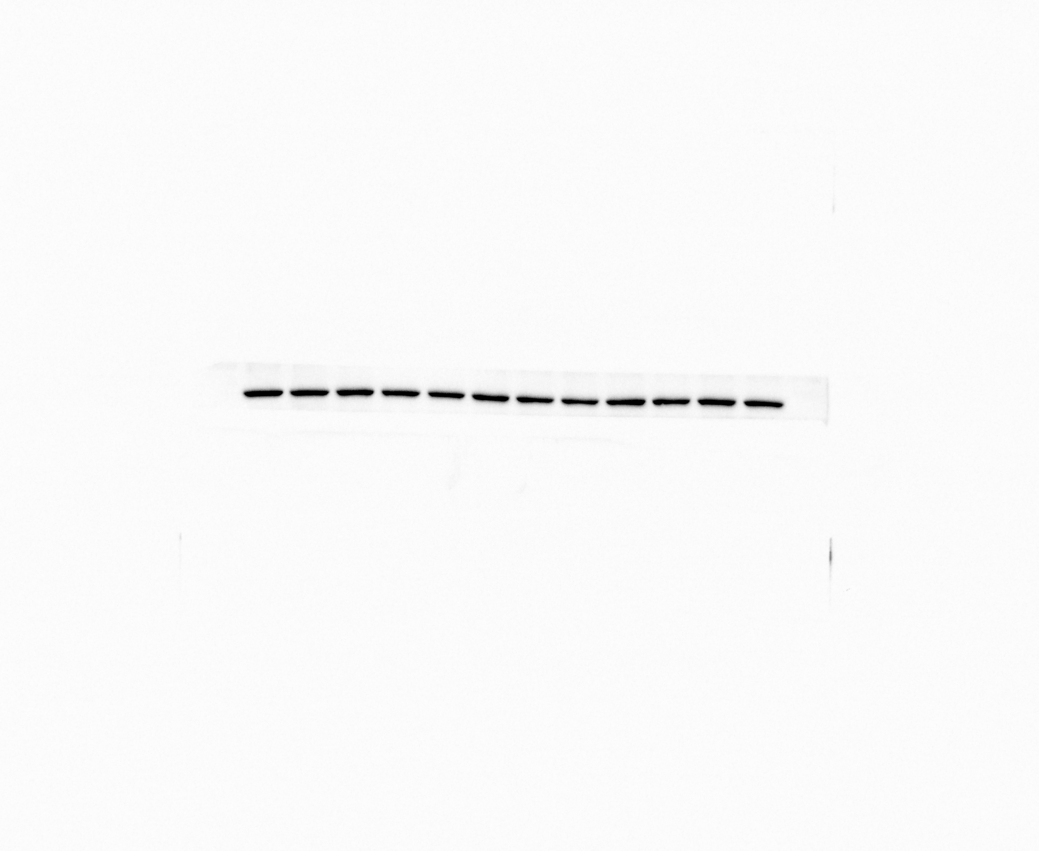


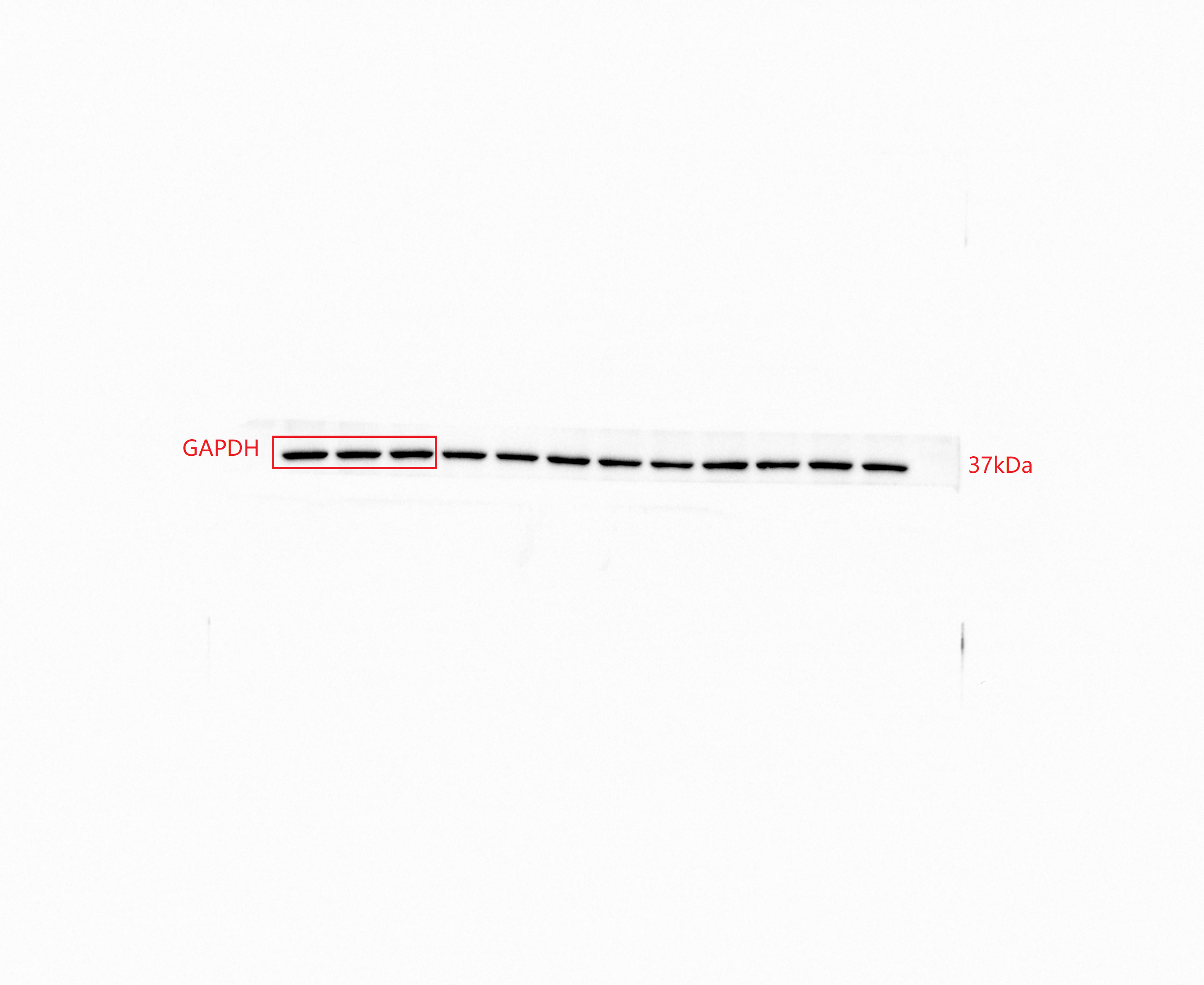


Figure 5 (GAPDH)

Supplement: Supplementary file 1 — Supplementary Material 1. [file 13048_2024_1392_MOESM1_ESM.docx]
